# Relationship Between Self-Reported Function, Functional Tests and Biomechanical Parameters in Patients 12 Months After Total Hip Arthroplasty: A Preliminary Cross-Sectional Study

**DOI:** 10.1007/s43465-023-00887-6

**Published:** 2023-04-09

**Authors:** Stefanie John, Michael Esch, Marvin Steinert, Kerstin Witte

**Affiliations:** 1 Department of Sports Science, Faculty of Humanities, Otto-von-Guericke-University, Magdeburg, Germany; 2 Department of Biomechanics, Faculty of Mechanical and Process Engineering, Offenburg University of Applied Sciences, Offenburg, Germany

**Keywords:** Total hip arthroplasty, PROM, Functional performance, Gait, Balance, Muscle strength

## Abstract

**Background/Purpose:**

Several methods are used to evaluate the outcome of total hip arthroplasty (THA), however, their relationship at different time points after surgery is unclear. The purpose of this exploratory study was to investigate correlations between self-report function, performance-based tests (PBTs) and biomechanical parameters in patients 12 months after THA.

**Methods:**

Eleven patients were included in this preliminary cross-sectional study. Hip disability and Osteoarthritis Outcome Score (HOOS) was completed for self-reported function. As PBTs, the Timed-up-and-Go test (TUG) and 30-Second-Chair-Stand test (30CST) were used. Biomechanical parameters were derived from analyses of hip strength, gait and balance. Potential correlations were calculated using Spearman correlation coefficient *r*.

**Results:**

HOOS scores and parameters of PBTs showed moderate to strong correlations (0.3 < *r* < 0.7). Correlation analysis between HOOS scores and biomechanical parameters revealed moderate to strong correlations for hip strength whereas correlations with gait parameters and balance were rather weak (*r* < 0.3). Moderate to strong correlations were also found between parameters of hip strength and 30CST.

**Conclusion:**

For THA outcome assessment 12 months after surgery, our first results indicate that self-report measures or PBTs could be used. Analysis of hip strength also appears to be reflected in HOOS and PBT parameters and may be considered as an adjunct. Given the weak correlations with gait and balance parameters, we suggest that gait analysis and balance testing should be performed in addition to PROMs and PBTs as they may provide supplementary information, especially for THA patients that are at risk for falls.

## Background

Total hip arthroplasty (THA) is considered a successful and effective procedure for patients with end-stage cox arthritis. Following THA, patients are overall satisfied with the operation results and report improvements in function and reduction in pain [1]. However, several studies have shown that asymmetries and deficits in strength, balance and range of motion were still present several months and even several years after the surgery [2, 3].

Therefore, it is important to have accurate and valid measurement instruments to evaluate the success of the operation as well as the recovery to detect potential persisting deficits. Several measurement tools and methods are used to assess the outcome of THA ranging from self-report questionnaires to performance-based tests and biomechanical examinations.

Patient-reported outcome measures (PROMs), which describe the functional ability and quality of life from the patient's perspective, have gradually become the main method for assessing joint replacement outcomes in clinical practice and clinical research [4]. Several PROMs are applied to assess general health and health-related quality of life (QoL) as well as provide information on pain and function of the affected hip joint. These include the Western Ontario and McMaster University Osteoarthritis Index (WOMAC), Medical Outcomes Study Short Form-36 (SF-36), Harris Hip Score (HHS) and the Hip and Osteoarthritis Outcome Scores (HOOS). The latter is an extension of the WOMAC and is considered particularly useful for assessing younger and more active patients following THA. Despite the widespread acceptance of PROMs due to cost-effectiveness and easy administration, the stand-alone use of PROMS remains controversial. PROMs are subjective parameters that may not be sensitive enough to detect postoperative changes in physical function as they are strongly influenced by psychological factors and pain [5, 6]. Moreover, PROMs are mainly used in clinical settings whereas therapists do not use PROMs but performance-based tests (PBT) to monitor progress and assess outcomes, such as the Timed-up-and-Go test (TUG) as well as Sit-to-Stand tests (STS) [7]. PBTs are easy to perform and can be used to assess functional strength of the lower extremities, static and dynamic balance, or mobility and fall risk. Especially after total joint arthroplasty, these tests can provide important objective information on functional performance and the progress of rehabilitation [7]. PBTs seem to be more sensitive for detecting functional impairments after hip replacement than PROMs. They provide information about the functional abilities, but not about the biomechanics of the movement and the causes of possible deficits [8]. This can only be achieved by objective biomechanical examinations, for example including analyses of gait, balance and lower extremity strength. From the results of biomechanical examinations, persistent muscle weaknesses or asymmetries of individual muscle groups between the operated and non-operated side of patients following THA can be specifically identified. For example, in the study by Judd et al., significant strength deficits were still found in the knee flexors and knee extensors one year post-THA. In contrast, performance-based tests (TUG) showed no significant differences between THA patients and control subjects [9].

Depending on the measurement environment (clinic, rehabilitation facility, research laboratory) one of the three examination methods (PROMS, PBTs, biomechanical examinations) is often preferred to evaluate the outcome after THA. Proper assessment of function is very important for patients following THA as data from registries have shown that low function after hip replacement is a risk factor for subsequent revision [10]. An increasing number of studies have studied the relationship between the different subjective and objective measures used to assess THA outcomes. Not all results are consistent, but many studies reported weak or no correlations between the patient-reported outcomes and objective parameters leading to the recommendation that both subjective and objective methods are needed for the evaluation of THA [7, 11]. Especially in the early clinical stages, discrepancies between PROMs and objective parameters were found [12]. While patients perceived improvements in physical function, objective parameters showed deterioration. It was concluded that PROMS should not be used as the sole criteria, especially in the early mobilization phase after surgery, as they may lead to an overestimation of functional capacity [12, 13].

There is some evidence that the correlations between subjective and objective measures may improve sometime after surgery, as the patient's perception of recovery and actual functioning may be more consistent [12]. However, there is no consensus yet and further research is necessary. In addition, previous studies only compared two evaluation methods, either PBT and PROMs or PROMs and parameters from biomechanical examinations. Therefore, the aim of this study was to include all three measurement methods and to investigate exploratively if there were correlations between the parameters of the three methods in THA patients 12 months after surgery.

## Materials and Methods

### Participants

Participants were recruited through calls in local newspapers. All participants were eligible if they had undergone unilateral primary THA approximately 12 months previously. Participants were excluded from the study if they already had another joint replacement or additional orthopedic or neurologic conditions that affected daily function. All participants gave written consent to participate in this study after being informed about the procedure, its purpose and possible risks linked to the participation. The study was approved by the local ethics committee of the Otto-von-Guericke-University Magdeburg (no. of vote: 04/22 on January 21, 2022) and carried out in line with the Declaration of Helsinki. It was registered in the German Registry of Clinical Trials under the ID: DRKS00028103.

### Measurement Protocol

For this cross-sectional study, the participants attended a single testing session, in which all measurements were performed. These included patient-reported function (HOOS), performance-based tests (TUG, 30CRT) and biomechanical examinations (analysis of gait, balance and hip muscle strength). The test methods were performed in the following order: HOOS, gait analysis, balance analysis, PBTs, and hip strength analysis. Prior to the test battery, demographic and anthropometric data were collected.

### Outcomes

#### PROMs

Patient-reported outcome was measured using the Hip Disability and Osteoarthritis Outcome Score (HOOS). This is a 40-item questionnaire divided into five subscales: Pain, Symptoms, Function in activities of daily living (ADL), Function in sports and recreation (Spo/Rec), and Hip-related QoL [14]. For each question, patients had to assign points ranging from 0 (never/ no difficulty) to 4 (always/extremely difficulty). A normalized score between 0 (worst level of function/worst well-being) and 100 (best level of function/best well-being) was calculated for each subscale. The HOOS has been proven a valid and reliable instrument for the assessment of hip-related functions and symptoms [14].

#### PBTs

Performance-based function was evaluated with the Timed-Up-and-Go test (TUG) and the 30-Second-Chair-Stand test (30CST). The TUG is a valid and reliable test for quantifying functional mobility in elderly persons [15] and has been widely used in patients following THA [7, 11, 12, 16]. The TUG measures the time it takes patients to stand up from a chair without the aid of their hands, walk three meters at a self-selected speed, turn 180°, and return to a sitting position [15].

The 30-Second-Chair-Stand test (30CST) is used to assess lower extremity strength. For patients, following THA it was found suitable and reliability was demonstrated [17] For the 30CST, the number of repetitions for standing up and sitting down from a chair is counted within 30 s. According to the protocol of Jones et al. patients are starting in a standardized sitting position with the back straight and with the arms crossed at the wrist and held against the chest [18]. At the start signal, patients were instructed to stand up and sit down again as many times as possible in 30 s. A repetition was valid if the patient's body was upright and straight when standing and returned correctly to the initial sitting position. Incorrectly executed stands were not counted.

Before the measurements of TUG and 30CST, correct execution of the tests was demonstrated by the tester and patients were given one practice trial for each test. Both tests were performed twice with a 1-min break in between, and the best values (lowest time duration for TUG and highest number of repetitions for 30CST) were used for further analyses.

#### Biomechanical Examinations

The biomechanical examinations included analyses of gait, balance and hip muscle strength.

To examine gait, spatial–temporal gait parameters were measured with the OptoGait system (OptoGait, Microgate, Bolzano, Italy). This floor-based photocell system consists of transmitting and receiving bars in which LEDs are implemented that communicate with each other. By detecting interruptions in the signal communication caused by the patient’s steps, gait events are calculated. The OptoGait system has been described as a valid and reliable tool for evaluating spatial–temporal gait parameters [19]. In this study, an 8-walkway was constructed with the bars placed parallel to each other at a distance of 1.5 m. Patients walked at a normal, self-selected speed through the 8-m corridor. They were instructed to cover a few meters before and after the actual test corridor to ensure a constant velocity without the acceleration and deceleration phase. Each patient completed 10 trials. Data of each patient were averaged across the 10 trials for further analyses. The outcome parameters included walking speed, step length asymmetry and contact time asymmetry. The two latter parameters were chosen to describe gait symmetry/asymmetry. Asymmetry was calculated using the difference between the right and left feet and expressed in percentage.

Static balance was assessed in the bipedal stance using a force plate (PLUX-Wireless Biosignals S.A, Lisbon, Portugal). For the bipedal stance, patients were asked to take off shoes and stand with both legs, hip-width apart, on the force plate with the arms hanging down at the sides. They were instructed to stand as quietly as possible. Two trials with a duration of thirty seconds were recorded. Balance data were sampled at 250 Hz and further processed using Matlab (Version 2018b, The Math-Works Inc., Natick, MA). The dataset was filtered by applying a 4th-order Butterworth low-pass filter with a 10 Hz cut-off frequency. As outcome parameters, the total length of the center of pressure (COP) was computed as well as the mean and maximum excursions of the COPx and COPy for mediolateral (ML) and anteroposterior (AP) directions [2]. The best trial (smallest COP length) was taken for further analyses.

**Fig.1:**
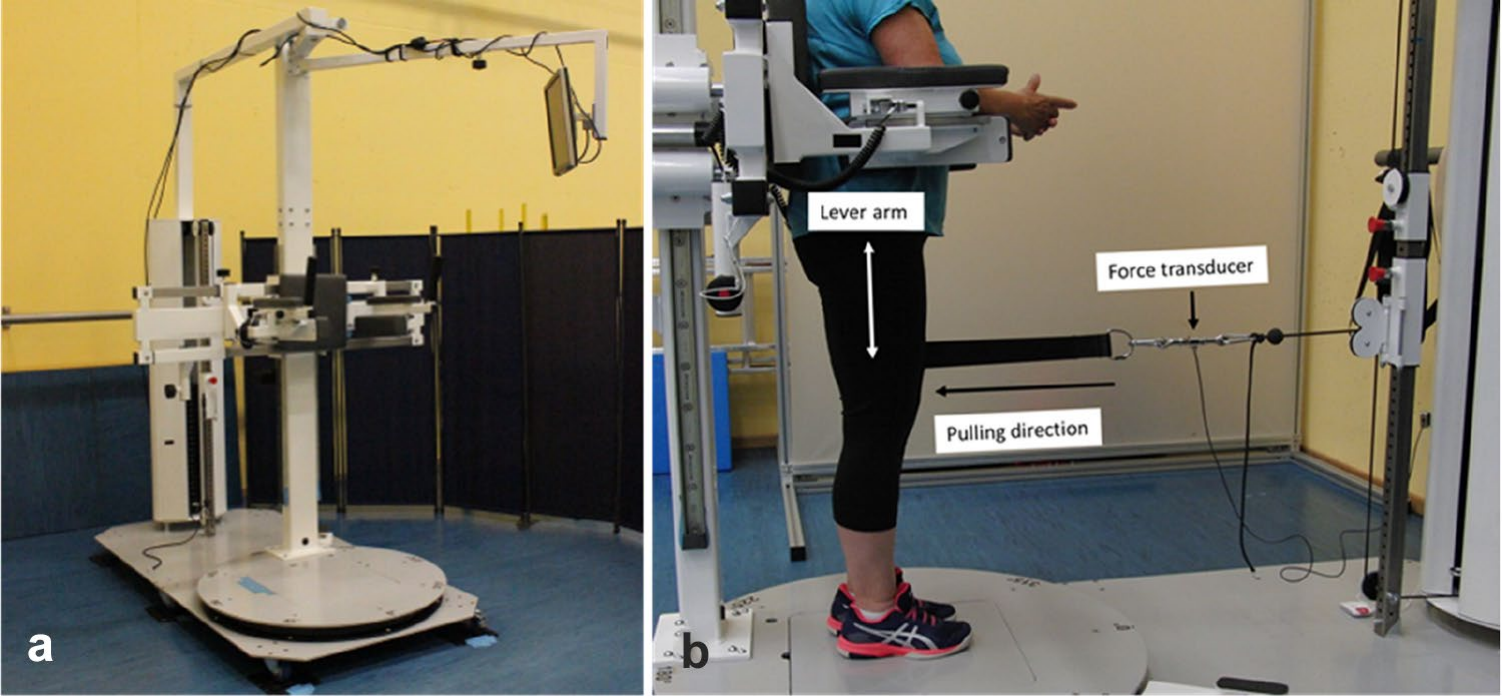
**a** Overall display of the diagnostic device, **b** Measurement set-up for measuring hip extension in the neutral hip position with the marked lever arm, the force transducer and pulling direction

The examinations of the isometric strength of the hip muscles were performed in a custom-made diagnostic device [2] (Fig. 1a). Patients were asked to stand in an upright position in the device. The pelvis support ensured that the participants remained in this position. A cuff was placed around the thigh serving as an attachment possibility for the hauling rope. An integrated force transducer (Hottinger Baldwin Messtechnik GmbH, Darmstadt, Germany) measured the isometric strength in the respective pulling directions of hip flexion, extension, abduction and adduction in the neutral hip position. In Fig. 1b, the measurement set-up for measuring isometric hip extension in the diagnostic device can be seen.

For each motion direction, one pretest and two main tests were performed. Participants were instructed to lift off the foot, build up strength and contract maximally without an abrupt push. A resting period of one minute between each trial was maintained. Hip strength data were obtained for both legs, the operated and the non-operated side. Force data from the strength analysis were acquired at 1000 Hz and filtered in Matlab with a 4th-order Butterworth low-pass filter (5 Hz). Torques were calculated using the distance between the greater trochanter and the point of applied force (the middle of the cuff) serving as the lever arm. Out of the two main trials, the maximum torque of each motion direction was extracted and normalized to the body mass of the participants. Maximum torques in hip flexion, extension, abduction and adduction of the operated side were used for further analyses as well as the average maximum hip torque of the operated side.

### Statistical Analyses

Statistical analyses were performed using IBM SPSS Statistics version 28 (IBM SPSS, Armonk, NY). Descriptive statistics were determined for all demographic, anthropometric and measurement variables. The relationship between patient-reported and performance-based outcomes as well as biomechanical variables was investigated using Spearman’s correlations coefficient *r*. Correlations were defined as weak (0.0–0.3), moderate (0.3–0.7) or strong (0.7–1.0) [20]. The significance level was set at *p* < 0.05.

## Results

Eleven patients (6 women and 5 men) with unilateral THA participated in the study. The mean post-operation time was 12 months. Further characteristics of THA patients are shown in Table 1.

**Table 1:** Demographic and anthropometric characteristics (mean ± SD) of THA patients

	THA patients (*n* = 11)
Age [years]	66.2 ± 4.9
Height [m]	1.68 ± 0.11
Mass [kg]	75.2 ± 16.4
BMI [kg/m^2^]	26.4 ± 3.7
Post-op time (months)	11.8 ± 5.5
Operated leg	5 × right, 6 × left
Indication for THA	
Coxarthrosis [*n*]	9
Trauma [*n*]	2

*BMI* body mass index; *SD* standard deviation

In Table 2, the results of HOOS scores, performance-based tests and biomechanical examinations of the THA patients are presented.

**Table 2:** Results of HOOS scores, performance-based tests and biomechanical examinations in THA patients

	THA patients
*HOOS score (0–100)*	
HOOS Symptoms	81 ± 16
HOOS pain	83 ± 15
HOOS ADL	82 ± 17
HOOS Spo/Rec	66 ± 15
HOOS QoL	66 ± 15
*Performance-based tests*	
TUG [s]	8.0 ± 1.2
30CST [*n*]	13.6 ± 2.3
*Gait*	
Walking speed [m/s]	1.33 ± 0.16
Step length asymmetry [%]	2.52 ± 1.81
Contact time asymmetry [%]	1.74 ± 1.40
*Balance bipedal stance*	
COP length [mm]	460.4 ± 154.9
Mean excursion ML [mm]	3.0 ± 1.3
Max excursion ML [mm]	15.5 ± 6.5
Mean excursion AP [mm]	5.8 ± 2.1
Max excursion AP [mm]	32.0 ± 12.6
*Hip strength*	
Flex op [Nm/kg]	1.55 ± 0.54
Ext op [Nm/kg]	1.05 ± 0.22
Abd op [Nm/kg]	1.11 ± 0.31
Add op [Nm/kg]	1.32 ± 0.37
Average hip strength [Nm/kg]	1.26 ± 0.54

*ADL* function in activities of daily living; *Spo/Rec* function in sports and recreation; *QoL* quality of life; *TUG* Timed-Up-and-Go test, *30CST* 30-Second Chair-Stand test; *COP* center of pressure; *ML* mediolateral; *AP* anteroposterior; *op* operated side

Table 3 shows the correlations between the HOOS subscale scores and the gait, balance, hip strength and PBT parameters. Concerning correlations between HOOS and biomechanical parameters, correlations varied from low to strong. A strong negative correlation was observed for the HOOS subscale Qol and step length asymmetry (*r* = – 0.71, *p* = 0.02). Besides this, no significant correlations were found between HOOS scores and gait parameters and HOOS scores and balance parameters.

**Table 3:** Correlations between HOOS subscale scores and parameters of gait, balance, hip strength and PBTs

	Spearman correlation *r*				
	HOOS Symptom	HOOS Pain	HOOS ADL	HOOS Spo/Rec	HOOS QoL
*Gait*					
Walking speed	0.26	0.58	0.34	0.28	0.43
Step length asymmetry	– 0.14	0.07	0.05	– 0.03	– 0.10
Contact time asymmetry	– 0.32	– 0.46	– 0.32	– 0.43	**– 0.71**
*Balance*					
COP length	– 0.01	– 0.04	– 0.20	0.21	– 0.33
Mean excursion ML	– 0.07	– 0.23	– 0.30	– 0.08	– 0.03
Max excursion ML	– 0.16	– 0.40	– 0.49	– 0.11	– 0.11
Mean excursion AP	0.22	0.35	0.12	0.59	0.09
Max excursion AP	0.25	0.19	0.05	0.48	0.33
*Hip strength*					
Flex op	0.30	**0.74**	0.59	0.52	0.56
Ext op	– 0.26	0.06	– 0.03	– 0.17	0.17
Abd op	0.19	0.49	0.41	0.13	0.58
Add op	0.25	**0.70**	0.45	0.49	0.43
Average op	0.19	**0.61**	0.48	0.38	0.48
*Performance-based tests*					
TUG [s]	– 0.42	**– 0.61**	– 0.28	– 0.52	– 0.48
30CST [n]	0.57	**0.77**	**0.64**	0.51	**0.72**

Significant correlations are marked in bold (*p* < 0.05)

*ADL* function in activities of daily living; *Spo/Re*c function in sports and recreation; *QoL* quality of life; *COP* center of pressure; *ML* mediolateral; *AP* anteroposterior; *op *operated side; *TUG* Timed-Up-and-Go test, *30CST* 30-Second Chair-Stand test

**Fig. 2:**
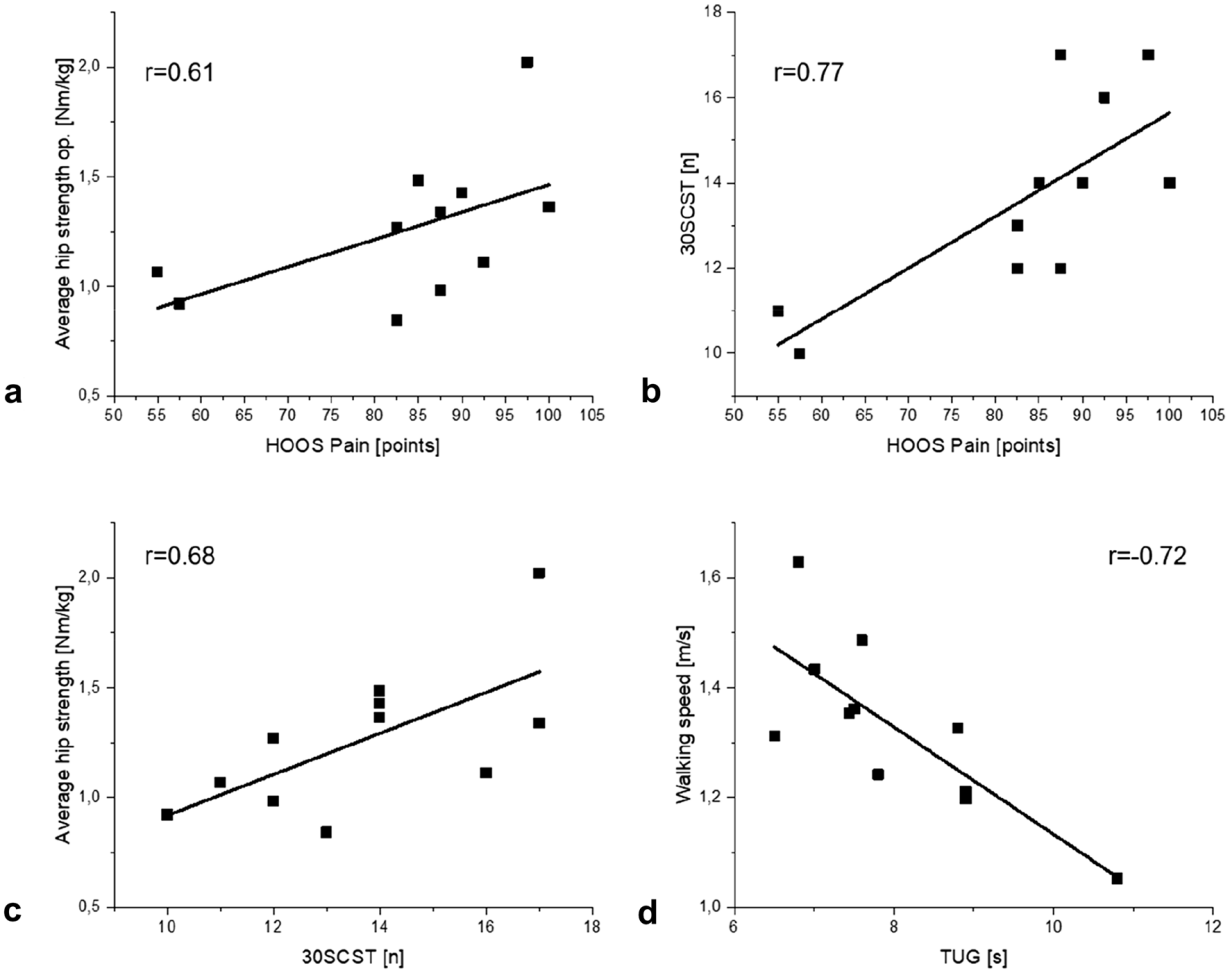
Correlations between **a** HOOS Pain and average hip strength, **b** HOOS Pain and 30CST, **c** 30CST and average hip strength and **d** TUG and walking speed

Several moderate to strong positive correlations were found between HOOS scores and hip strength. Significant correlations were observed between the HOOS subscale Pain and hip flexion (*r* = 0.74, *p* = 0.01), hip adduction (*r* = 0.71, *p* = 0.02) and average hip strength (*r* = 0.61, *p* = 0.04). The latter correlation is visualized in Fig. 2a).

Concerning the relationship between HOOS score and results of PBTs, almost all HOOS subscales showed moderate to strong correlations with TUG and 30CST. For the HOOS subscales Pain, ADL and QoL significant correlations were revealed in particular with the 30CST (Pain: *r* = 0.77, *p* = 0.01; ADL: *r* = 0.64, *p* = 0.03; Qol: *r* = 0.72, *p* = 0.01). As an example of the correlation of HOOS with PBTs, the correlation between HOOS Pain and 30CST is shown in Fig. 2b).

The relationship between the outcome of the performance-based tests and hip strength, balance and gait parameters was also investigated. The correlations can be seen in Table 4. The 30CST was significantly correlated with almost all hip strength parameters with the correlation coefficients ranging between 0.6 and 0.7 indicating moderate to strong correlations. The correlation between 30CST and average hip strength is presented in Fig. 2c).

**Table 4:** Correlations between outcomes of PBTs and biomechanical parameters in THA patients

	Spearman correlation *r*	
	TUG	30CST
*Gait*		
Walking speed	– **0.72**	0.24
Step length asymmetry	0.32	0.30
Contact time asymmetry	0.12	– 0.45
*Balance bipedal stance*		
COP length	0.04	– 0.33
Mean excursion ML	0.28	– 0.15
Max excursion ML	0.28	– 0.41
Mean excursion AP	– 0.12	0.13
Max excursion AP	0.06	0.03
*Hip strength*		
Flex op	– 0.37	**0.64**
Ext op	– 0.06	0.36
Abd op	– 0.43	**0.71**
Add op	– 0.47	**0.77**
Average op	– 0.27	**0.68**

Significant correlations are marked in bold (*p* < 0.05)

*TUG* Timed-Up-and-Go test; *30CST* 30-Second Chair-Stand; *COP* center of pressure; *ML* mediolateral; *AP* anteroposterior; *op* operated side

The TUG showed a negative relationship with hip strength with moderate correlations. However, none of them were statistically significant.

Similarly, no significant correlations were found between PBTs and balance and gait parameters, with the exception of walking speed. A strong negative correlation was detected with TUG (*r* =  −0.72, *p* = 0.01), which is shown in Fig. 2d).

## Discussion

For the evaluation of the recovery and success of THA surgery, different criteria are used including self-report measures, outcomes of performance-based tests and biomechanical parameters whose relationship at different time points after THA is not clear. In this study, we included all three different evaluation methods and investigated potential correlations between them in THA patients 12 months after surgery. The correlation analysis between HOOS scores and objective parameters revealed moderate to strong correlations for hip strength whereas correlations with gait and balance were rather weak. Concerning the relationship between HOOS scores and parameters of PBTs, moderate to strong correlations were found.

The relationship between PROMs and PBTs has been investigated and discussed in literature before. Especially in the early postoperative period, weak correlations have been described between HOOS scores and PBTs, such as TUG, Stair-Climbing test and 30CST [12, 21] Due to the discrepancy between these two methods, the combined use of both methods is recommended during the early clinical stages to avoid overestimation of patient functional capacity [7, 11]. In our study, however, moderate correlations between HOOS scores and TUG and even strong correlations between some subscales of HOOS and 30CST were found in THA patients 12 months after surgery. Similar results were also reported in the study of Elibol et al., in which the relationship between PROMs and PBTs was examined in THA patients 4 years after surgery. Moderate to strong correlations were found between subscales of SF-36, HHS and PBTs as TUG and Sit-to-Stand tests [22]. As suggested by Dayton et al., the relationship between PBTs and PROMs seems to get better over time as the patient's perception of recovery and actual functioning may be more consistent [12]. Our findings support this assumption. The combined use of PBTs and PROMs is recommended for THA patients in the early postoperative period, but for follow-up examinations 1 year after surgery or later, our results suggest that PROMs or PBTs can be used for assessment.

In contrast to PBTs, biomechanical examinations are less included in THA outcome assessments and only a few studies investigated potential correlations with PROMs. We found moderate to strong correlations between HOOS scores and hip muscle strength. To the author’s knowledge, this is the first study to include an analysis of hip strength for possible correlation with PROMs. One study was found that investigated this relationship in patients following total knee arthroplasty. Furu et al. examined correlations between quadriceps strength and PROMs one year after joint replacement and stated that higher quadriceps strength was associated with improved PROMs [23], which is in line with our results.

Other studies, that included biomechanical parameters mostly used sensor-based gait parameters as objective parameters. Just as observed in our study, rather weak relationships were reported with PROMs [24, 25]. In the study of Bolink et al., weak correlations were found between WOMAC scores and several gait parameters such as speed, cadence, and step asymmetry in THA patients 12 months after surgery [25]. Similar results were reported by Boekesteijn et al., who examined THA patients 15 months after joint replacement [24]. The authors found that objective gait parameters showed a different postoperative recovery trajectory than HOOS scores, and concluded that gait parameters may provide additional information about the physical function.

Given the weak correlations between PROMs and gait and balance parameters found in our study, we assume that balance and gait diagnostics, as performed in our study, are not reflected in HOOS parameters. We suggest that gait analysis and balance testing should be performed in addition to PROMs, especially in THA patients who have difficulty walking or are at a specific risk for falls.

Biomechanical examinations, however, usually need lots of time and capacity that may not be always available. For this reason, we also looked into potential correlations between PBTs and parameters from gait, balance and hip strength analysis. Moderate to strong correlations were found between PBTs and hip strength parameters. Significant positive correlations were especially found for the 30CST, which was to be expected as the 30CST is a demanding test used to assess lower extremity strength [18]. Our results indicate that this test is a good indicator of hip strength and could therefore replace biomechanical hip strength analysis. For the TUG, however, which is supposed to rather reflect gait and balance abilities, no significant correlations were found with gait and balance parameters, except walking speed. The TUG has been recommended as a screening tool, especially for frail elderly patients [15]. However, for a more detailed analysis, we suggest that biomechanical analyses of gait and balance should be performed.

Some limitations have to be addressed. The major limitation of this study is the small sample size of THA patients. The results of the correlation analyses should be interpreted with caution and cannot be generalized. This study had more of an exploratory character to investigate the relationship between different subjective and objective outcome parameters 12 months after THA. Future studies with a larger sample size should also consider potential factors that may influence outcome parameters such as diagnosis, surgical approach, and type of implant. Another limitation is that the study was only cross-sectional. To draw a conclusion about the extent to which PBTs or biomechanical parameters provide more information than PROMs, it is necessary to conduct a longitudinal study from before surgery to one year after surgery, documenting changes in parameters and examining how these changes are correlated between the three methods.

This study included three different methods for THA outcome assessment and investigated potential correlations in patients 12 months after THA. Due to moderate to strong correlations between HOOS and parameters of PBTs in both methods, our first results indicate that for THA evaluation one year post-op, both methods could be used. Concerning the biomechanical parameters, the hip strength also appears to be reflected in HOOS and PBT parameters and may therefore be considered as an adjunct. Given the weak correlations with gait and balance parameters, we suggest that gait analysis and balance testing should be performed in addition to PROMs and PBTs as they may provide more information, especially in THA patients who have difficulty walking or are at a specific risk for falls.

These preliminary findings need to be confirmed in future studies with a larger sample size.
